# Complications of Primary Realignment of Posterior Urethral Disruption After Pelvic Trauma

**DOI:** 10.5812/traumamon.13523

**Published:** 2014-03-01

**Authors:** Mohammad Haidari, Alireza Azargoon, Hormoz Mahmoudvand, Vahid Almasi, Yadollah Pournia, Manouchehr Shams Khorramabadi

**Affiliations:** 1 Department of Urology, Lorestan University of Medical Sciences, Khorramabad, IR Iran; 2 Department of Internal Medicine, Lorestan University of Medical Sciences, Khorramabad, IR Iran; 3 Department of Surgery, Lorestan University of Medical Sciences, Khorramabad, IR Iran; 4 Department of Medical Education, Clinical Research Center, Lorestan University of Medical Sciences, Khorramabad, IR Iran; 5 Department of Language, Lorestan University of Medical Sciences, Khorramabad, IR Iran; 6 Department of Nursing, Lorestan University of Medical Sciences, Khorramabad, IR Iran

**Keywords:** Urethral Stricture, Erectile Dysfunction, Urinary Incontinence

## Abstract

**Background:**

There are two fundamental selections for the management of traumatic posterior urethral injury, delayed repair or early primary realignment.

**Objectives:**

The aim of this study was to assess the complications of primary realignment of posterior urethral disruption.

**Patients and Methods:**

This retrospective descriptive cross-sectional study was done at the Shohada-ye Ashayer University Hospital in Khorramabad. All male patients admitted to the hospital with posterior urethral disruption and had undergone primary realignment of the urinary tract between 2003 and 2010 were included. Primary realignment of the urinary tract was done up to 24 hours after injury. The patients underwent open cystostomy and then a nelaton catheter was inserted from the bladder neck to the distal urethra anterogradely. Upon voiding from the catheter, another nelaton catheter was fixed to it and was pulled into the bladder. The catheter was removed if the urethra was intact in the retrograde urethrography after three weeks. The patients were followed for six months. The data were presented as mean and percentage.

**Results:**

A total of 24 patients were evaluated while seven, eleven, four, and two patients were aged under 20, 20 to 39, 40 to 59, and over 60 years old, respectively. Thirteen patients (54.16%) had urinary tract stenosis after the primary realignment. Erectile dysfunction was reported in three of them. Urinary incontinence did not occur in patients without stenosis.

**Conclusions:**

Early primary realignment of posterior urethral disruption had significant complications. In this study we did not have a control group, thus we could not compare the complications of delayed repair and early primary realignment of the posterior urethra. We recommend further case-control studies with larger sample size.

## 1. Background

Approximately 4 to 14% of pelvic fractures lead to posterior urethral rupture (1, 2) that can cause urethral stricture, urinary incontinence, and erectile dysfunction (2). There are two fundamental selections for the management of traumatic posterior urethral injury: Suprapubic tube placement with delayed repair (3-6 months later), or early primary realignment (1-15 days later) (3). However, there is controversy over the timing and management methods for managing traumatic posterior urethral injury (4). Some studies reported that delayed repair has more problems compared with early primary realignment.

## 2. Objectives

The aim of this study was to assess the complications of primary realignment of posterior urethral disruption.

## 3. Patients and Methods

This retrospective descriptive cross-sectional study was done at the Shohada-ye Ashayer Hospital of Lorestan University of Medical Sciences in Khorramabad in 2011. All 24 male patients admitted with posterior urethral disruption and had undergone primary realignment of the urinary tract from April 2003 to February 2010 were included. In these patients, posterior urethral disruption was diagnosed with a retrograde urethrogram. Primary realignment of the urinary tract were done up to 24 hours after injury. Mainly the patients underwent open cystostomy and then a 14-F nelaton catheter was inserted from the bladder neck to the distal urethra anterogradely. When voiding was seen from the catheter, another 14-F nelaton catheter was fixed to it and pulled into the bladder. All the operations were performed by one urologist. After three weeks, the urethra was evaluated with a retrograde urethrogram. The contrast agent was injected to the urethra beside the catheter and if the urethra was intact in the retrograde urethrography, the catheter was removed. Afterwards, we blockaded the cystostomy catheter and when there were no urinary abnormalities, the cystostomy catheter was removed. All patients received one gram of cephalothin every six hours for one week. When there was no further indication for hospitalization, the patient was discharged after 24 hours. The patients were followed-up for six months. The patients’ data and required information regarding subsequent surgical procedures and therapeutic outcomes were obtained from hospital records and by means of telephone contacts with patients. To diagnose impotence, the patients were asked about history of night and morning erections and sexual activities. Finally, the data were presented as mean and percentage. 

## 4. Results

Posterior urethral rupture in all 24 patients was caused by blunt pelvic trauma. The patients were in the age range of 7 to 75 years old. They were divided into four age groups of under 20, 20 to 39, 40 to 59, and over 60 years old. A total of seven patients (29.16%) were aged under 20 years, 11 patients (45.8%) were in the age group of 20 to 39, four patients (16.7%) were in the age group of 40 to 59, and two patients (8.33%) were older than 60 years old. Thirteen patients (54.16%) had urinary tract stenosis after the primary realignment including six patients (46.2%) in the age group of under 20, four patients (30.8%) in the age group of 20 to 39 , two patients ( 15.4%) in the age group of 40 to 59, and one patient ( 7.7%) in the age group of over 60 years old. Among patients with urinary tract stenosis, six of them (46.1%) underwent open urethroplasty, four patients (57.1%) underwent urethrotomy once, two patients (28.6%) underwent urethrotomy twice, and one of them underwent open surgery after urethrotomy (twice) due to the recurrence of the stenosis. Furthermore, erectile dysfunction was reported in three patients (12.5%) in the age groups of 40 to 59 and over 60 years old. In our study, urinary incontinence was not seen in the patients without stenosis. However, it was observed in severe stressful conditions in two patients with stenosis after primary repairing, who had undergone internal urethrotomy. 

## 5. Discussion

The usual method for the management of traumatic posterior urethral injury is delayed urethroplasty. However, some studies reported that, compared with early primary realignments, this method has some problems. A prior study reported that, in the children, the progression of urethral stricture in early primary realignment was less than that in the delayed method (5). Hence, they suggested early primary realignment for managing the injuries of the posterior urethra in children. In addition, another study declared that the results of early realignment might be better than the results of delayed open urethroplasty for the management of posterior urethral disruption (6). In our study, 24 patients with posterior urethral disruption underwent early primary realignment of the urinary tract from April 2003 to February 2010. Thirteen patients (54.16%) had urinary tract stenosis after the primary realignment. The frequency of urinary tract stenosis in the age group of less than 20 years old was more than the rates in other age groups (85.71%). The prevalence of this disorder was 46.7% in patients aged between 20 and 59 years. A previous study reported that 24% of the 18 to 70 years old patients that underwent primary realignment of the urinary tract in Shiraz (south of Iran) experienced postoperative stricture (7). Furthermore, in another study (50%) of the children that underwent primary realignment of the urinary tract in India required additional urethrotomy for managing the stricture (8). Moreover, it was reported that urethral stricture developed in 16.6% of the children that underwent primary realignment of the urinary tract in Turkey. (5) Also, another study stated that postoperative stricture occurred in 49% of the patients that underwent primary realignment (6). The frequency of postoperative strictures in our study was more than the rates in all the above-mentioned studies. In our study, erectile dysfunction was reported in three cases (20%) of patients aged 20 to 59 years. While erectile dysfunction developed in 16% of patients in a prior study (7), its frequency in our study was higher but was less frequent than that in a study that declared 33.6% of patients had erectile dysfunction (6). This difference may be due to the long follow-up period in that study (average of 8.8 years). In addition, another study reported that 36% of the patients that underwent primary realignment had erectile dysfunction (3). In our study, urinary incontinence did not occur in the patients without stenosis. However, it was observed in severe stressful conditions in two patients with stenosis after primary repairing, who had undergone internal urethrotomy. Regarding urinary incontinence in patients who underwent primary realignment, previous studies reported it to occur in none (7), 17.7% (6), and 8.6% (9). Our study did not include a control group. Thus, we could not compare the complications of delayed repair and early primary realignment of the posterior urethra. Further studies with larger sample size are recommended.
